# A parsimonious characterization of change in global age-specific and total fertility rates

**DOI:** 10.1371/journal.pone.0190574

**Published:** 2018-01-29

**Authors:** Athena Pantazis, Samuel J. Clark

**Affiliations:** 1 Department of Sociology, The University of Washington, Seattle, United States of America; 2 Department of Sociology, The Ohio State University, Columbus, United States of America; 3 MRC/Wits Rural Public Health and Health Transitions Research Unit (Agincourt), School of Public Health, Faculty of Health Sciences, University of the Witwatersrand, Johannesburg, South Africa; 4 ALPHA Network, London School of Hygiene and Tropical Medicine, London, United Kingdom; 5 INDEPTH Network, Accra, Ghana; Hokkaido University Graduate School of Medicine, JAPAN

## Abstract

This study aims to understand trends in global fertility from 1950-2010 though the analysis of age-specific fertility rates. This approach incorporates both the overall level, as when the total fertility rate is modeled, and different patterns of age-specific fertility to examine the relationship between changes in age-specific fertility and fertility decline. Singular value decomposition is used to capture the variation in age-specific fertility curves while reducing the number of dimensions, allowing curves to be described nearly fully with three parameters. Regional patterns and trends over time are evident in parameter values, suggesting this method provides a useful tool for considering fertility decline globally. The second and third parameters were analyzed using model-based clustering to examine patterns of age-specific fertility over time and place; four clusters were obtained. A country’s demographic transition can be traced through time by membership in the different clusters, and regional patterns in the trajectories through time and with fertility decline are identified.

## Introduction

**Fig 1:**
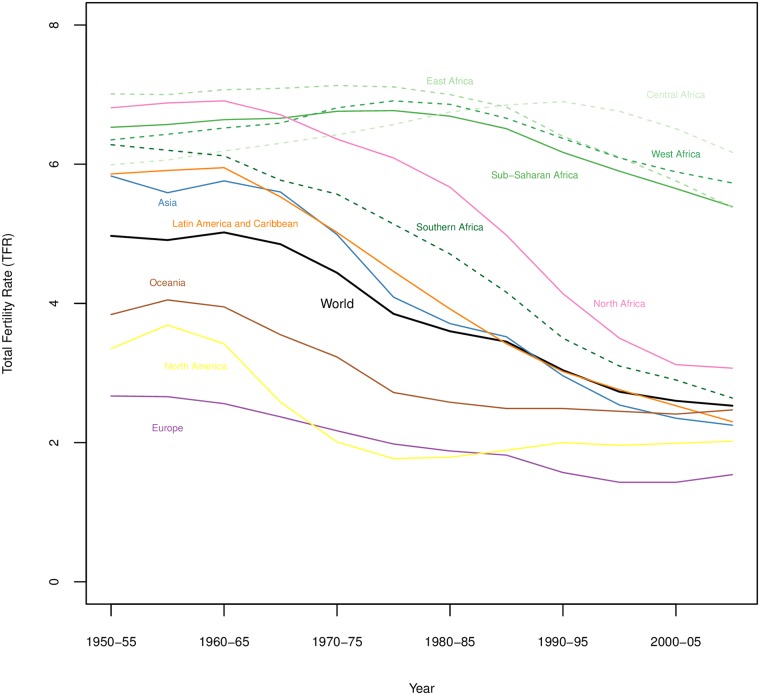
Trends in TFR by world region, with sub-regions for sub-Saharan Africa. Data from UN World Population Prospects [6].

Fertility change remains an area of concern for demographers and policymakers in large part due to the continued rapid growth of the world population, set to reach 9.7 billion by 2050 with over 50% of growth occurring in sub-Saharan Africa (SSA), predominantly driven by slow fertility decline [1]. Demographic transition theory posits that once begun fertility decline should be relatively steady and irreversible, and most demographers agree that fertility decline had begun virtually everywhere by the late 1980s [2] or the 1990s [3], even in SSA. However, SSA fertility has remained high and stalls in fertility decline have been identified in some countries (for example see [4]) posing a challenge for reconciling SSA fertility trends with what demographic transition theory would predict. Fig 1 shows trends in total fertility rates (TFR) for the world and world regions from 1950-55 until 2005-10, showing the decline across all regions, though with substantially different slopes. Analysis of the convergence of fertility has demonstrated that fertility levels around the globe are converging, which is suggested in Fig 1, though high fertility in SSA has been an outlier in global fertility convergence for decades [5].

**Fig 2:**
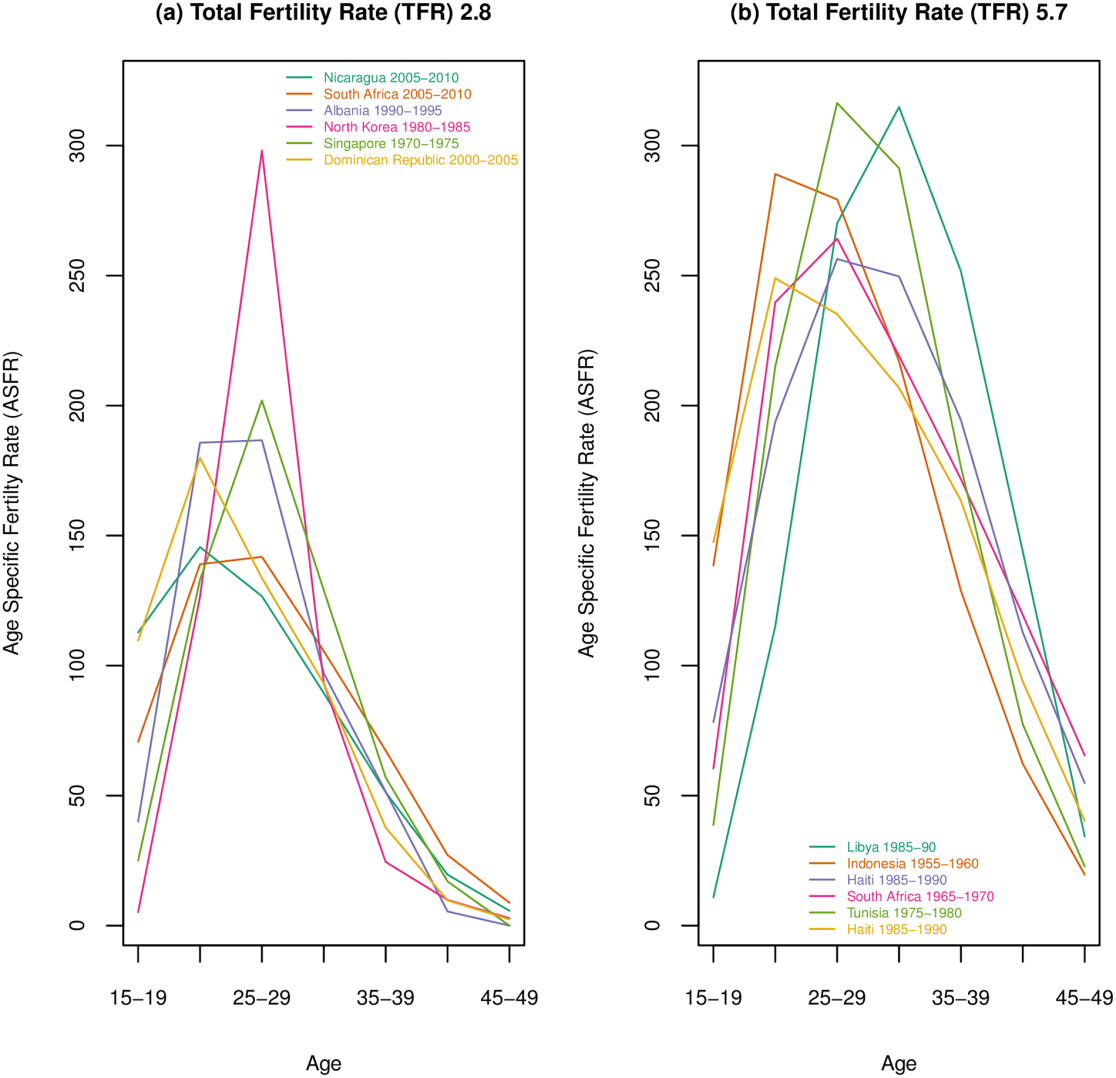
Example age-specific fertility curves producing the same total fertility rate. The first panel shows a selection of age-specific fertility curves that create a total fertility rate of 2.8; the second panel shows different curves that sum to a total fertility rate of 5.7. Data from UN World Population Prospects [6].

While most literature that seeks to characterize and understand fertility transition focuses on the TFR, a single number for a place and time, the TFR is built from age-specific fertility rates (ASFR). ASFRs compose the fertility regime that is under analysis when one looks at the TFR. However, very different age patterns of fertility can produce the same TFRs, as can be seen in Fig 2, and the age pattern of fertility, and how it changes, is important for understanding fertility change in a population. In the first panel of Fig 2 six age-specific fertility curves corresponding to a TFR of 2.8 are shown. These curves are quite distinct from one another, with some curves, such as the Dominican Republic, Singapore and North Korea being very peaked, with fertility concentrated in a single age group, while others such as South Africa and Albania have highest fertility spanning at least 2 age groups. In the second panel curves corresponding to a TFR of 5.7 are shown, and these curves vary even more widely, with some starting with very high fertility at youngest ages and others having relatively high late fertility. Generally, fertility decline created through the control of fertility has been hypothesized to decline at older ages first, followed by a decline at the youngest ages. Knodel found evidence of this pattern in Europe and in most of Asia [7]. Applying this pattern to Fig 2, one would theorize that South Africa 1965-70 and Indonesia 1955-60 have started decline and seen a drop in older age fertility, perhaps, while Libya and Tunisia still have high fertility at all ages. It would be difficult to assess Haiti’s curve, which is relatively low at youngest ages but high at older ages. The decline in Latin America, generally, did not have a consistent pattern of changes in ASFRs, with countries falling broadly into three categories of ASFRs at the beginning of fertility decline, defined as early peak, late peak and dilated peak by Chackiel and Schokolnik [8], with many countries maintaining the age structure they had at the beginning of decline. Other countries changed over time, transitioning from dilated to early peak, late to early peak, and others from a late to dilated peak [8]. Caldwell and colleagues argue that fertility decline in SSA will occur in a very different way compared to Asia and the West, due to different constraints on premarital and extramarital sexuality, differences in marital stability, and different emphases on the need and reasons for birth spacing; and they hypothesize that in SSA the fertility decline will occur at all ages [3]. Van de Walle and Foster [9] compared SSA fertility patterns to patterns seen in Western and Asian fertility declines, particularly marked decline at oldest ages and/or highest parities, and found “considerable uncertainty about the causes and permanence of these trends”, indicating, at least in part, that SSA was not exhibiting the patterns associated with early fertility decline seen by Knodel [7] or others. More recent work by Moultrie and colleagues [10] has found widening birth intervals at all ages and parities associated with fertility decline in SSA, supporting Caldwell and colleagues’ [3] claims of differences in the SSA decline, at least in part. However, questions remain about how SSA age-specific fertility curves fit into a global context.

The question our approach is seeking to address is, can we find commonalities in *how* age-specific fertility changes across world regions and across time, and do these patterns provide insight into the future of fertility in areas with persistent high fertility? This paper seeks to address primarily the first portion of this question by applying a novel approach to investigating age-specific fertility over time and across countries and searching for patterns that can be associated with fertility decline.

This paper aims to present an analytical approach to understanding global fertility trends from 1950-2010 utilizing age-specific fertility rates, from the UN World Population Prospects. To create a quantitative chasracterization of fertility schedules for comparison through time and across countries, we consider the ASFRs rather than the TFR. This approach acknowledges the different ASFRs that may aggregate to similar TFRs but mean something different about fertility transition or decline. This approach is similar to methods used in previous work by the authors and their colleagues [11–14] and makes use of singular value decomposition (SVD) to decompose age-specific fertility curves and reduce the number of parameters needed to describe them.

Other methods have been used to investigate age patterns of fertility. Coale and Trussell’s [15] model of age-specific fertility rates based on first marriage patterns and martial age-specific fertility and estimates of m, their parameter capturing the magnitude of difference between observed and model marital fertility schedules, has been used to determine the level of fertility control in a population. However, this approach is focused on marital fertility, which, given the data available and importance of non-marital fertility in the most recent decades, is of limited interest in understanding recent fertility trends or for making fertility projections. The Lee-Carter method for fertility projection, based on their mortality projection method, applies SVD to the age-specific residual remaining after subtracting mean age-specific fertility [16]. Their approach is similar to the one used in this paper but our approach is importantly different. We do not mean-subtract the age-specific fertility rates; we compute the SVD of the age-specific data directly; and we use more than the first component because of our interest in better understanding the changes in age-specific fertility over time, rather than building a projection model for the TFR, as was the aim of Lee’s fertility models. Hyndman and Ullah [17] build on the Lee-Carter method, extending it to functional data by conducting a functional PCA on smoothed data and using ARIMA models for the forecasting. A major difference in our approach is that we are including fertility regimes from multiple countries over time, instead of looking at just one country’s regimes. We are not smoothing our data in any fashion, though the data are already smoothed, somewhat, as these fertility data undergo some manipulation before publication by the UN.

We begin by describing the data we will be using in this analysis. Next we will describe the SVD method used as applied to age-specific fertility curves. Then we will present an illustrative example of this approach using data from Sweden. Then we will show results from applying this method to fertility curves through time for 154 countries and discuss global patterns identified using this approach. Finally, we conclude with a discussion of our findings.

## Materials and methods

### Data

Data used for this analysis come from the UN’s World Population Prospects (WPP) 2012 Revision [6]. ASFRs are provided for all countries in five year intervals from 1950-55 to 2005-10 for five year age groups (ages 15-19 to ages 45-49). Countries with a population of at least one million were included in the analysis; 154 countries met this criterion. Three countries, Yemen, Gabon and Timor-Leste, had outlier TFR trajectories that suggested problems with their data or extremely exceptional fertility trends, so they too were excluded. Six age-specific fertility rates are used (age 15-19 years, 20-24 years, 25-29 years, 30-34 years, 35-39 years, and 40-44 years) for the 12 time periods. The last age group, ages 45-49, was omitted from this analysis because the fertility rates for that age group were close to zero, or were zero, and inclusion greatly influenced the results of the analysis at the expense of details related to fertility at earlier and higher fertility ages. Because our model operates on the full real line and fertility rates are positive, logged ASFRs are used for the analysis. A consequence of this is that the values we model are all negative which influences the interpretation of our results. All visualizations show exponentiated results, however, so as to be directly comparable to age-specific fertility curves on their natural scale.

Data from the UN WPP are the most complete data available for global fertility and provide data for 50 years for all countries in the world, despite the fact that these data are already processed, and to an extent, smoothed by the UN. No other data cover as much time for the globe. Additionally these are the same fertility data used by the UN Population Division to inform their global estimates and population projections, and thus finding patterns and understanding the trends in these data will be useful for informing predictions of fertility. These same data have been used in comparative analyses of fertility trends elsewhere as well, for example [5, 18, 19]. These data are available directly from the UN WPP website: http://esa.un.org/unpd/wpp/.

An illustrative example is presented below using data from the Human Fertility Collection [20]. Swedish data provide annual age-specific fertility from 1891-2011 for each age 15-50 years. These data were chosen for illustrative purposes because of their high quality over a duration that covers high fertility through decline to replacement and the period of below-replacement fluctuations, thus allowing a full view of fertility change by year. These data are also available online: http://fertilitydata.org.

### Methods

This section summarizes the material in Clark [13] which presents this method and its application in full detail.

The singular value decomposition (SVD) (e.g. [21]) factorizes a matrix **X** such that

$$X=USV^{\text{T}}\;,$$ (1)

where **U** contains the orthonormal (independent, unit length) ‘left singular vectors’ **u**_i_ (columns of **U**), **V** contains the orthonormal (independent, unit length) ‘right singular vectors’ **v**_i_ (columns of **V**), and **S** is a diagonal matrix containing the ‘singular values’, denoted as *s*_i_.

The right singular vectors are a new set of orthonormal dimensions for the points defined by the rows of **X**. The product of the left singular vectors and their corresponding singular values are the projections of the points defined by the rows of **X** along the new dimensions defined by the right singular vectors.

The SVD is estimated by minimizing the distance between the actual points (rows of **X**) and the best approximations of those points using successively more of the new dimensions defined by **V**. The singular values correspond to the fraction of the overall squared distance from the origin to the points along the new dimensions **V** that is captured by each individual new dimension **v**_i_. The first new dimension is oriented to capture as much of this perpendicular squared distance as possible, and each successive new dimension captures the most possible of what remains.

The product of the SVD factors can be algebraically rearranged to yield another equivalent expression called the *Eckart-Young-Mirsky formula* [22]

$$X=\sum_{i=1}^{\rho }s_{i}u_{i}v_{i}^{\text{T}}\;.$$ (2)

Eq (2) expresses **X** as a sum of rank-1 matrices, where *ρ* is the rank of **X**. By construction (above) the first term in this sum captures or explains the bulk of the variation in the original data (rows of **X**), and each subsequent term explains less and less. The expression for **X** in Eq (2) can be further rearranged to express each column vector **x**_*ℓ*_ in **X** as

$$x_{\ell }=\sum_{i=1}^{\rho }s_{i}v_{\ell i}u_{i}\;.$$ (3)

Eq (4) says that we can write all the columns in **X** as weighted sums of the left singular vectors scaled by their corresponding singular values. The weights are the *ℓ*^th^ elements of each corresponding right singular vector. Moreover, the Eckart-Young-Mirsky matrix approximation theorem [22] reveals that these sums have the property of concentrating most of the variation in the first few terms, and consequently we only need the first few terms to produce approximate values for **x**_*ℓ*_ that are very close to the actual values. This allows us to closely approximate the columns of **X** with (potentially very) few effective parameters—just the first few weights.

Using the SVD, the 6 × 1,848 (age × country, time) matrix of ASFRs from the UN WPP data is factored into *i* ∈ {1, …, 6} orthogonal age-varying components *s*_i_ ⋅ **u**_i_ (the left singular vectors scaled by their corresponding singular values, six elements each, one for each age group) and country-time-varying weights associated with those components, the 1,848 elements of each **v**_i_.

**Fig 3:**
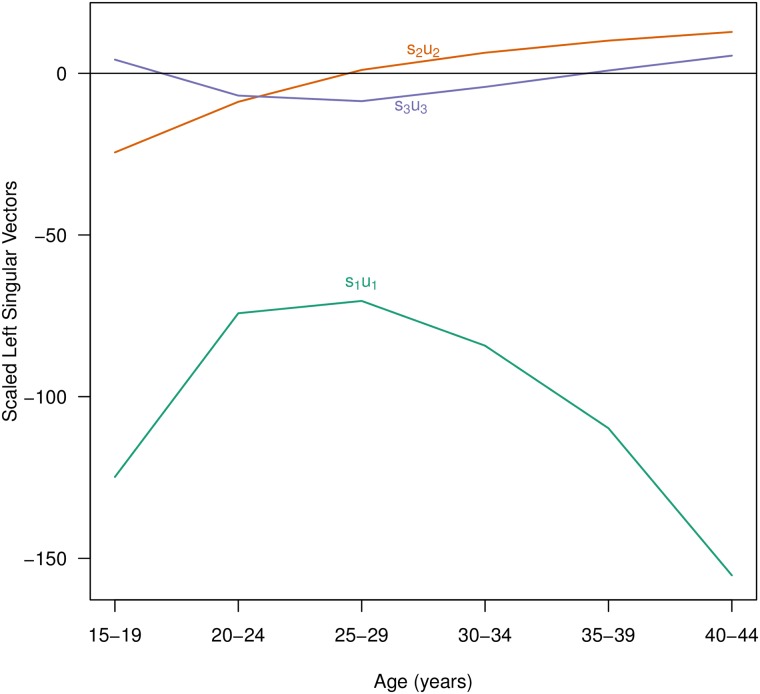
First three components (scaled left singular vectors) from SVD factorization of age-specific fertility schedules. Data from the UN WPP [6] for 154 countries in five year intervals from 1950-55 to 2005-10 for five year age groups.

Following [13], the first component *s*_1_ ⋅ **u**_1_ is the underlying ‘shape’ of the age-specific fertility schedules (*s*_1_**u**_1_ in Fig 3), and the remaining components define increasingly subtle refinements to that underlying shape. As the Eckart-Young-Mirsky matrix approximation theorem suggests, adding additional components, or terms to the sum in Eq (4), adds increasingly more refined but less consequential nuances to the reconstructed fertility schedule, until when all components are included, the reconstruction is equal to the original. In this application we are retaining the first three components, *s*_1_**u**_1_, *s*_2_**u**_2_ and *s*_3_**u**_3_, shown in Fig 3. *s*_1_**u**_1_ can be seen to have the general shape an age-specific fertility curve, while *s*_2_**u**_2_ decreases (or increases) early or late fertility and *s*_3_**u**_3_ accentuates how peaked or flat the curve is. The adjustments made by the second and third components are relatively subtle refinements (dependent on the magnitude of their **v**_i_ values) but provide important differentiation between age-specific fertility curves over time and across countries.

The first three left and right singular vectors and singular values closely approximate the majority of the 1,848 empirical fertility schedules included in the matrix originally factored using the SVD. Consequently, the model of age-specific fertility that we manipulate is

$$f_{ct}=\sum_{i=1}^{3}v_{ict}\cdot s_{i}u_{i}\;,$$ (4)

where **f**_ct_ is the age-specific fertility schedule for country *c* in time period *t*, and *i* indexes the three SVD-derived age-specific components that we retain. The columns of the original data matrix **X** are country and time-specific; hence each column is identified by a unique combination of *c* and *t*.

The SVD factorization allows us to work with a high quality, three-parameter approximation of the full age-specific fertility schedules. Beyond having fewer dimensions, the SVD factorization produces effective parameters, the *v* weights, that are independent and interpretable because they are associated with fixed age-specific components whose age profiles are meaningful.

1. *v*_1_ controls the overall level of fertility: as *v*_1_ increases, fertility decreases (because we model logged fertility rates)
2. *v*_2_ controls the comparative levels of young and old fertility: as *v*_2_ increases fertility shifts to older ages
3. *v*_3_ controls how concentrated fertility is near the middle of the childbearing years, or how ‘peaked’ the age profile of fertility is: as *v*_3_ increases, the age profile of fertility becomes less concentrated in the middle childbearing years.

We describe how the weights (elements of the right singular vectors) change through time by country and region, and using the mclust model-based clustering method, we group the two-element ‘weight vectors’ associated with each age-specific fertility schedule into clusters of similar weight vectors, and hence similar fertility schedules. Model-based clustering is conducted using the mclust package in R [23] on the second and third weights selected from the SVD results. Bayesian Information Criteria (BIC) is used to select the optimal number of clusters. Each resulting cluster has its own characteristic age pattern of fertility.

## Results

### Illustrative example with data from Sweden

**Fig 4:**
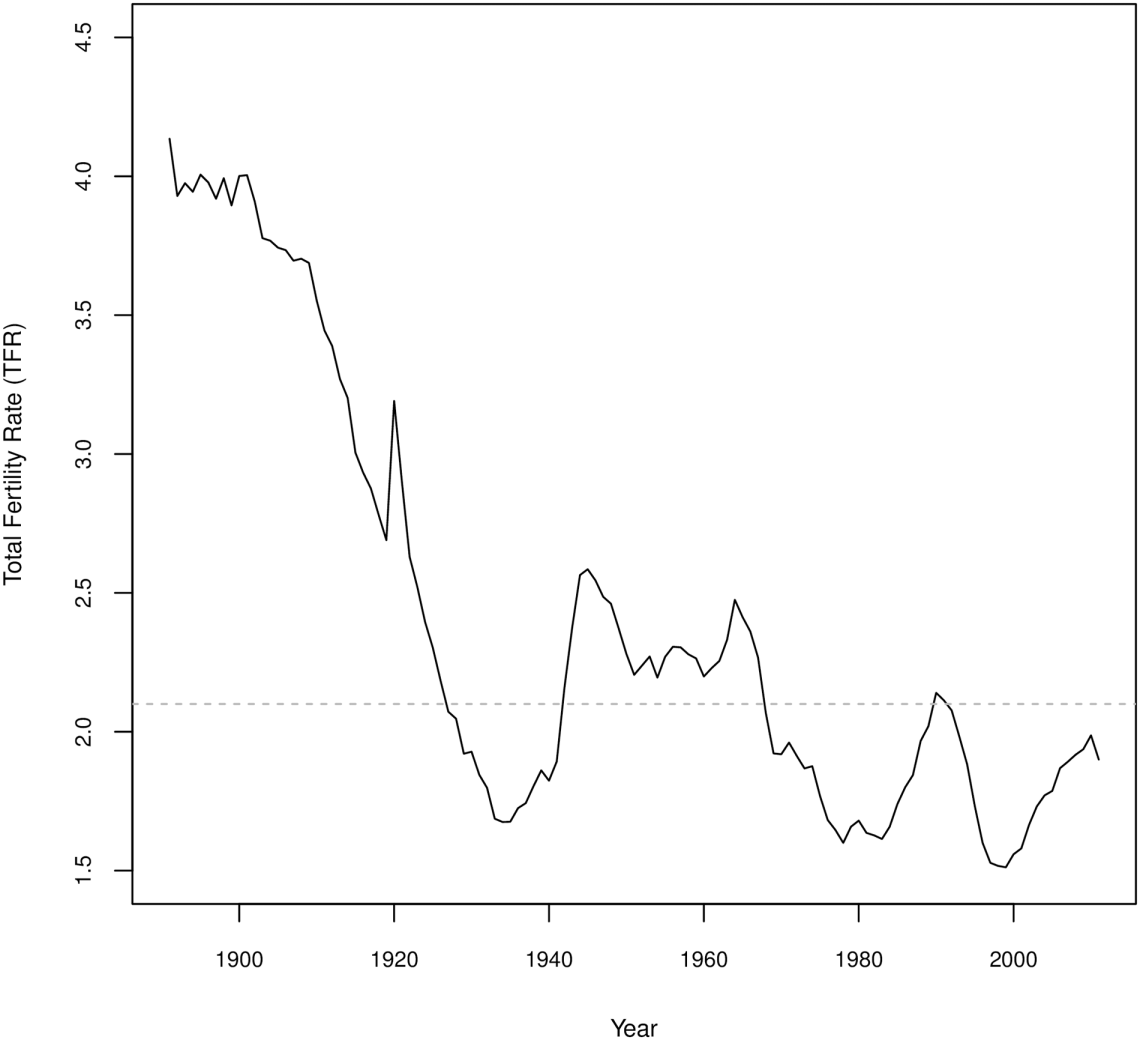
Total fertility rate for Sweden from 1891 to 2011. Data from the Human Fertility Collection [20].

Data for Sweden available from the Human Fertility Collection [20] cover high fertility in the 1890s through Sweden’s initial fertility decline, baby boom and post-transition, low fertility years, thus providing a time series that covers nearly all of Sweden’s fertility decline (data are not available for the highest levels of fertility and so there is no data included for fertility levels near pre-transition levels seen in non-Western regions). Fig 4 shows Sweden’s TFR over time for the data used.

**Fig 5:**
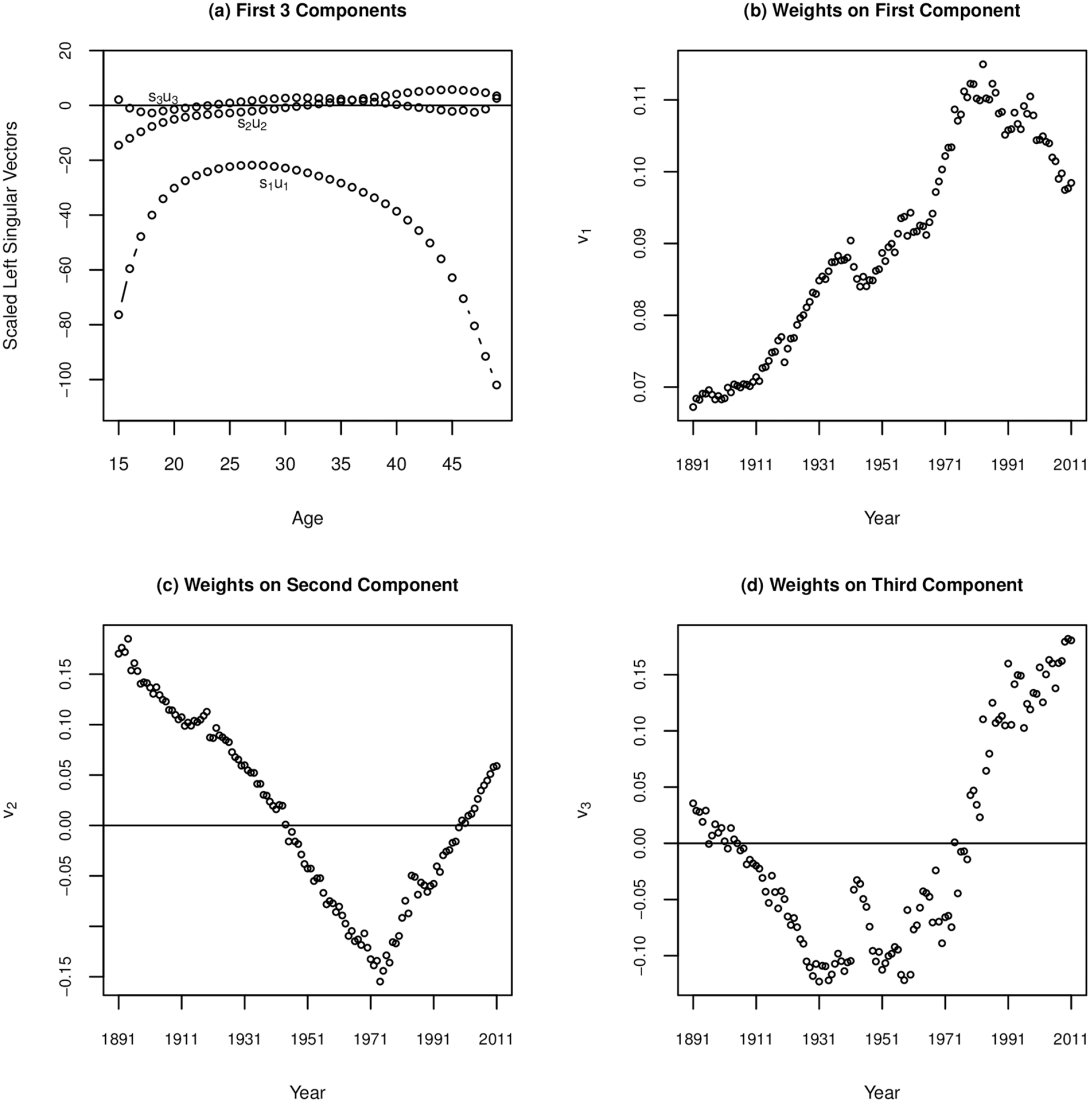
SVD results for Swedish ASFR 1891-2011. (a) Scaled left singular vectors, showing the first 3 of 35 components from the SVD; (b) Weights (*v*_1_) associated with the first component (*s*_1_**u**_**1**_); (c)Weights (*v*_2_) associated with the second component (*s*_2_**u**_**2**_); (d) Weights (*v*_3_) associated with the third component (*s*_3_**u**_**3**_).

Using the Swedish data, we obtain similar components from the SVD (see Fig 5(a)) as we see in Fig 3 for the UN WPP data. The main difference is that the curves are much smoother because age is in single years. Additionally, we see even more nuance in the second and third components. For the UN WPP data, we retain three out of six total components, however for Sweden we achieve a similar level of detail retaining *three out of 35 components* (more total components are obtained for Sweden because the initial matrix is 121 years by 35 ages). Component two is primarily functioning to accommodate lower early fertility and higher later fertility (or the opposite for weights of opposite sign) though we see some nuance in how this is affecting the oldest age groups, seeing that at the oldest age group’s fertility is not changing. The third component has a similar structure here as for the UN WPP data, though more nuance through the 20s and 30s, the ages of highest fertility across this data.

Panel b in Fig 5 shows how the weights on the first component change with time. Generally this weight is increasing, which corresponds to a decline in overall fertility levels, and peaks in the 80s and 90s before declining in the most recent years, which is consistent with the TFR over those years. Additionally we can see breaks in the pattern of declining fertility corresponding to World War II and the baby boom, and to a lesser extent World War I and the global flu pandemic of 1918.

Panels c and d in Fig 5 show the weights for the second and third components over time. Of note is that even for these more nuanced components we can see visible impacts of historical events. The weights on the second component have a noticeable increase around the end of World War I and the 1918 flu, with a less distinct one occurring around the end of World War II, indicating a relative increase in fertility at later ages (or a shift in the peak fertility ages to slightly older ages). Otherwise the weights are declining regularly from the beginning of the data through the early 1970s passing through zero, which indicates shifts to lower fertility at older ages and a shift of peak fertility to younger ages. From the 1970s, the trend reverses completely, with a steady increase in the values of the weights, even passing into positive values again, though there is a notable blip around the late 1980s. Increases in the values of *v*_2_ are associated with a shift towards higher fertility in later years. The third component similarly starts declining from the beginning of time under study, which corresponds to a flattening of the age-specific fertility curve. However, third component weights are quite volatile from the late 1930s through the 1960s, taking on a strange shape. Following 1960, these weights incline steadily, corresponding to fertility being concentrated in fewer ages, or a more pronounced peak in the curve. Likely, the trends seen starting in the late 60s for these weights are associated with the availability of modern contraception profoundly shaping fertility behaviors.

### Results for global fertility trends from 1950-55 through 2005-10

#### Results from SVD

Fig 3 plots the shapes of the age-specific curves of the first three components, denoted as *s*_i_**u**_i_. The first component captures the overall shape of the curve of fertility with age, rising steeply from the first age group to a peak in the second through fourth age groups, and steadily declining thereafter. The weights on *s*_1_**u**_1_ are all positive. Larger weights on *s*_1_**u**_1_ result in lower fertility, pulling the curve down, and smaller weights result in higher fertility, pushing the curve up.

The second component adjusts the earliest and oldest age groups, to accommodate higher or lower early or late fertility, while the third component adjusts the peak fertility age, in the 20s and early 30s, either flattening the curve or intensifying the peak. Positive weights for the *s*_2_**u**_2_ curve bring fertility down in the earliest age groups while simultaneously pulling up fertility at later ages, leaving fertility levels at the second age group unchanged (the opposite would occur with a negative weight on *s*_2_**u**_2_, pulling up earliest fertility while suppressing later fertility and effectively shifting peak fertility to younger ages (negative) or to older ages (positive)). Similarly, depending on the sign of the weight on *s*_3_**u**_3_, combining this curve with *s*_1_**u**_1_ accentuates/de-accentuates peak fertility, pushing up the fertility rates at the peak ages while suppressing fertility at the extreme ages. Subsequent components made further, more complex and more subtle adjustments to the basic curve given by *s*_1_**u**_1_.

#### SVD weights over time

**Fig 6:**
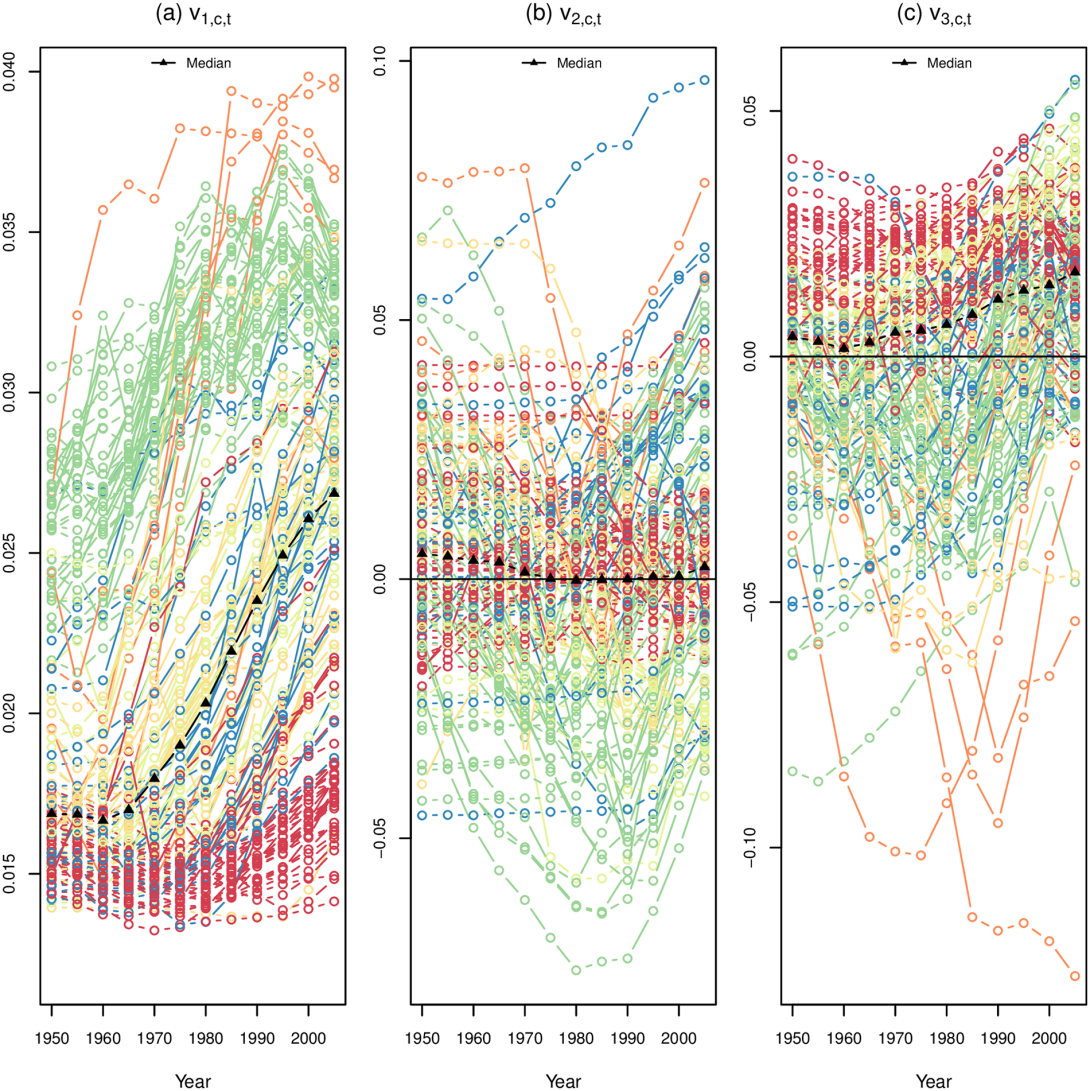
Weights on each component by country from 1950-55 through 2005-2010. (a) Shows the first component weights, *v_1ct_*; (b) shows the second component weights, *v_2ct_*; and (c) shows the third component weights, *v_3ct_*. Colors indicate world region: red for sub-Saharan Africa, orange for East Asia, yellow for South Asia, light green for Latin America and the Caribbean, green for the West, and blue for the Middle East and North Africa.

The country-time specific SVD-derived weights, *v_ict_*, are a parsimonious description of country-specific changes in age-specific fertility rates. Panel (a) in Fig 6 shows the weights for the first component for each country over time, with the color indicating the world region. Higher values of *v_1ct_* are associated with lower overall fertility and the median curve shown in black shows that values of *v_1ct_* have been steadily increasing globally since the 1960s. Though world regions do overlap, we can see distinctly that SSA countries are predominantly at the bottom of the plot, with the lowest values of *v_1ct_* through time and with the smallest increase over time. The West, comprised of the US, Canada, Australia, New Zealand and Europe, had some of the highest values for this weight in the earliest years and has maintained, after a rise in the 1960s and 1970s, high values for this weight. For East Asia we can see a dramatic increase from low levels to the highest values of *v_1ct_*.

Weights on the second component are largely centered around zero and the variance is much smaller than seen for *v_1ct_*, in Panel (b) of Fig 6. Looking at the way *v_2ct_* changes over time for each country, colored by world region, we do not see the regional patterns that are present in the relationship between *v_1ct_* and time. However, SSA countries seem highly concentrated near zero, in contrast to other regions that experience more variation in values of *v_2ct_* over time. This clearly reveals the lack of age-specific fertility change in SSA compared to other world regions. Tracking individual country lines through time, there appear to be substantial changes in values of *v_2ct_* period to period as well as a wide variety of patterns for overall time trends. Some patterned behavior can be seen for both East Asia and the West, while most other world regions exhibit far less variability and less dramatic change over time.

Weights on the third component, *v_3ct_*, over time for each country are shown in Fig 6 Panel (c). Like the second component, these weights are largely concentrated around zero and regional patterns for changes in *v_3ct_* over time do not seem particularly pronounced, though SSA countries seem concentrated just to the positive side of zero, while East Asia and the West seem to have much higher volatility in values and predominantly negative values for *v_3ct_*. As the third component is responsible for how peaked the fertility curves are, we see that SSA values are associated with flatter curves, or curves with peaks that span over multiple age groups, while the West and especially East Asia have curves that are quite peaked with highest fertility occurring in only one age group. This corresponds to general patterns of low overall fertility in these areas, one to two births, often relatively close together while in SSA the highest fertility years span more ages as women tend to have more than two children with sometimes substantial spacing [2].

#### Clustering results

**Fig 7:**
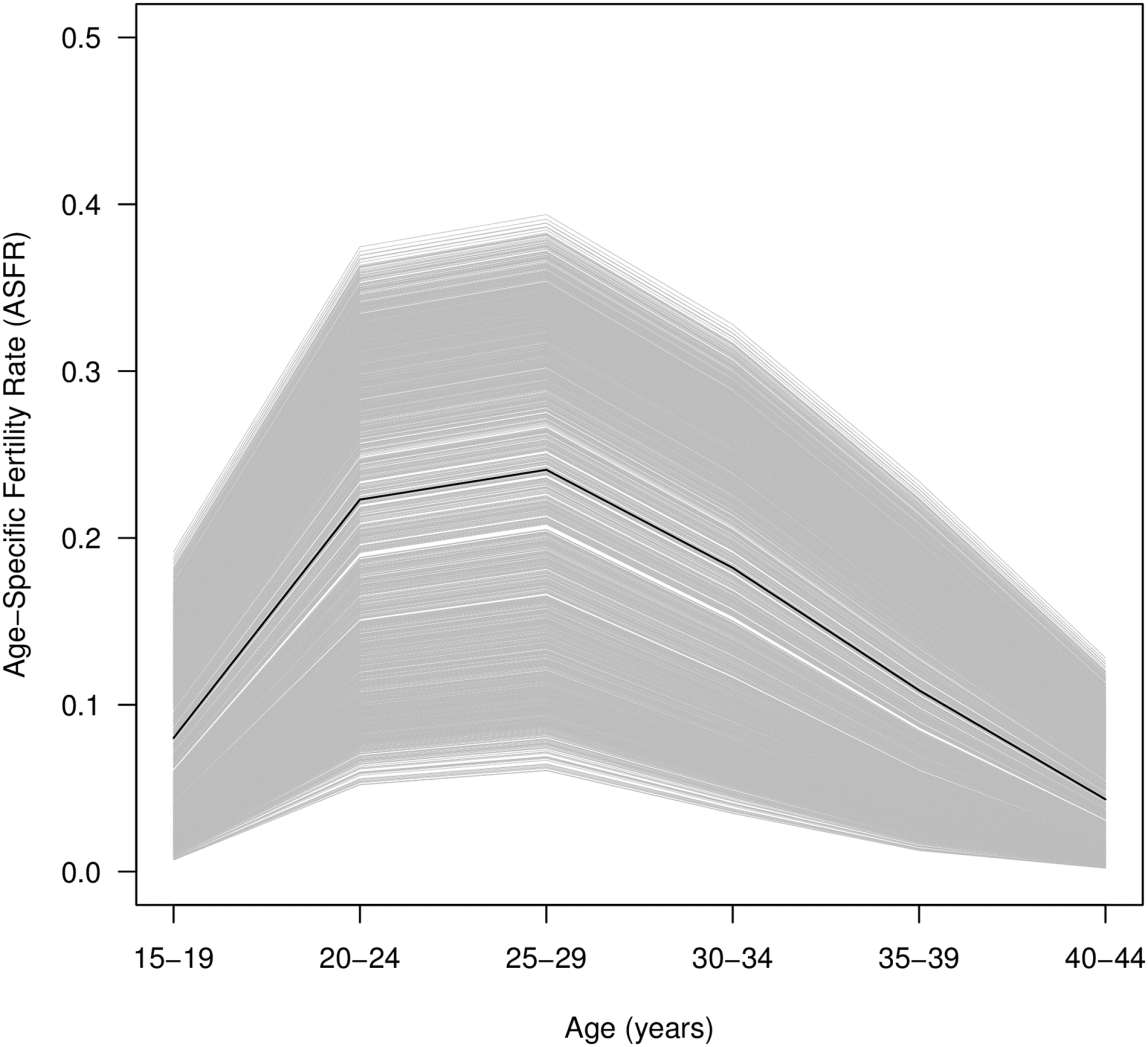
Age-specific fertility curve reconstructed from first component only. Median age-specific fertility curve constructed from the first SVD component only shown in black. All curves constructed for each county and time period shown in gray.

**Fig 8:**
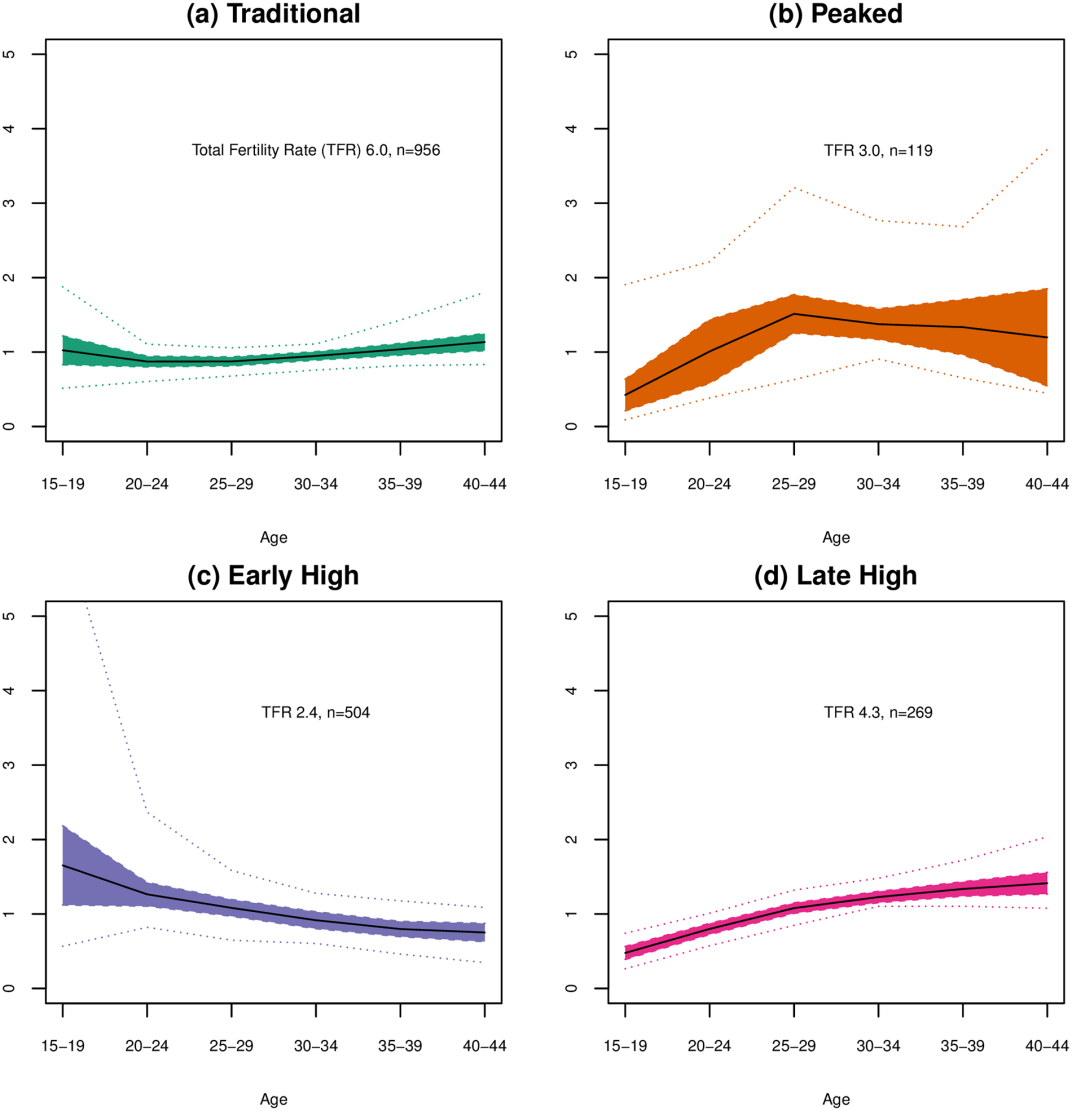
Changes to general age-specific fertility curve by cluster. Median exponentiated reconstructed curve is shown with interquartile range shaded and dotted lines showing extrema values for combined changes to the base age-specific fertility curve by including weighted components 2 and 3 for each of the 4 clusters: (a) the Traditional Curve cluster; (b) Peaked cluster; (c) Early High cluster; and (d) Late High cluster. These curves show the cluster characteristic deviations from the first component curve; values of 1 represent no change to the base curve.

The weighted first component describes the overall level on an age-specific fertility curve. The exponentiated *v_1ct_*⋅*s*_1_**u**_1_ curves for the 1848 country-periods included in the data is shown in Fig 7. This curve shows the age-specific fertility curve reconstructed from only the first component and the curve created using the median values for *v_1ct_* is shown in black. As seen above, this component functions much like TFR, capturing fertility levels. To explore how, independent of fertility level, age patterns of fertility may be similar or different across time and place, we have clustered the weights for the second and third components, which capture differences in young and old fertility and how peaked the fertility curve is. Four clusters of similar age-specific fertility curves were obtained from clustering vectors of the second and third weights. The reconstructed, exponentiated curves obtained are shown in Fig 8, which shows the median curves created by looking at the weighted second and third components for each cluster, with the interquartile range shaded and extrema values shown as dotted lines. Effectively, these curves show the variation seen in that cluster net of the basic age-specific fertility curve provided by the first component.

The Traditional cluster has the most classic curve, with minimal variation from the curve created by the first component. This cluster has the highest median fertility, with a TFR of 6, but has curves with a wide range of TFRs, from 0.9 to 8.3. In this cluster, peak fertility occurs at ages 25-29 years, and highest fertility spans ages 20 through 34 years. Fertility is relatively low in the youngest ages and declines steadily and gradually through the 30s and 40s, with fertility at the oldest age group lower than that of the youngest age group. Most SSA curves are in this cluster, and all world regions have curves in this cluster. The Traditional cluster was the largest cluster with 956 age-specific fertility regimes.

The Peaked cluster diverges from the basic curve obtained by the first component with a distinctive peak in the middle reproductive years after a rather sharp increase from low early fertility and a relatively sharp decline afterwards. The median curve places this distinct peak at ages 25-29, though curves in this cluster may have earlier peaks, later peaks or peaks that span more than one age group. This cluster had the fewest members, 119 fertility regimes, though there is substantial variation in the shape of these curves despite the unifying peak. The median TFR for these curves is 3.0 but TFRs ranged from 1.0 to 8.0 in this cluster. This cluster contains no curves from Latin America and the Caribbean or from SSA.

The Early High cluster is named for its higher fertility at early ages. The median TFR for its 504 member curves was 2.4, over a range of 1.2 to 7.6. The median curve for this cluster peaks at ages 20-24 and then declines steadily. Generally, these curves have a slight decrease or even an increase in fertility from ages 20-24 to ages 25-29 followed by a gradual decline to the oldest ages (some with very slow decline). Curves from all regions except East Asia are represented in this cluster.

The Late High cluster is named for its higher fertility at older ages. These curves are similar to those in the Traditional cluster, but while the curves in the Traditional cluster have fertility concentrated at and before ages 25-29 years, in this cluster’s curves fertility is concentrated in ages 25-29 and 30-34 years. Also, fertility at the youngest ages is lower in this cluster than in the Traditional cluster. TFRs for these curves range from 1.2 to 8.4 for the 269 member curves, and median TFR is 4.3. No curves from Latin America and the Caribbean are included in this cluster.

**Fig 9:**
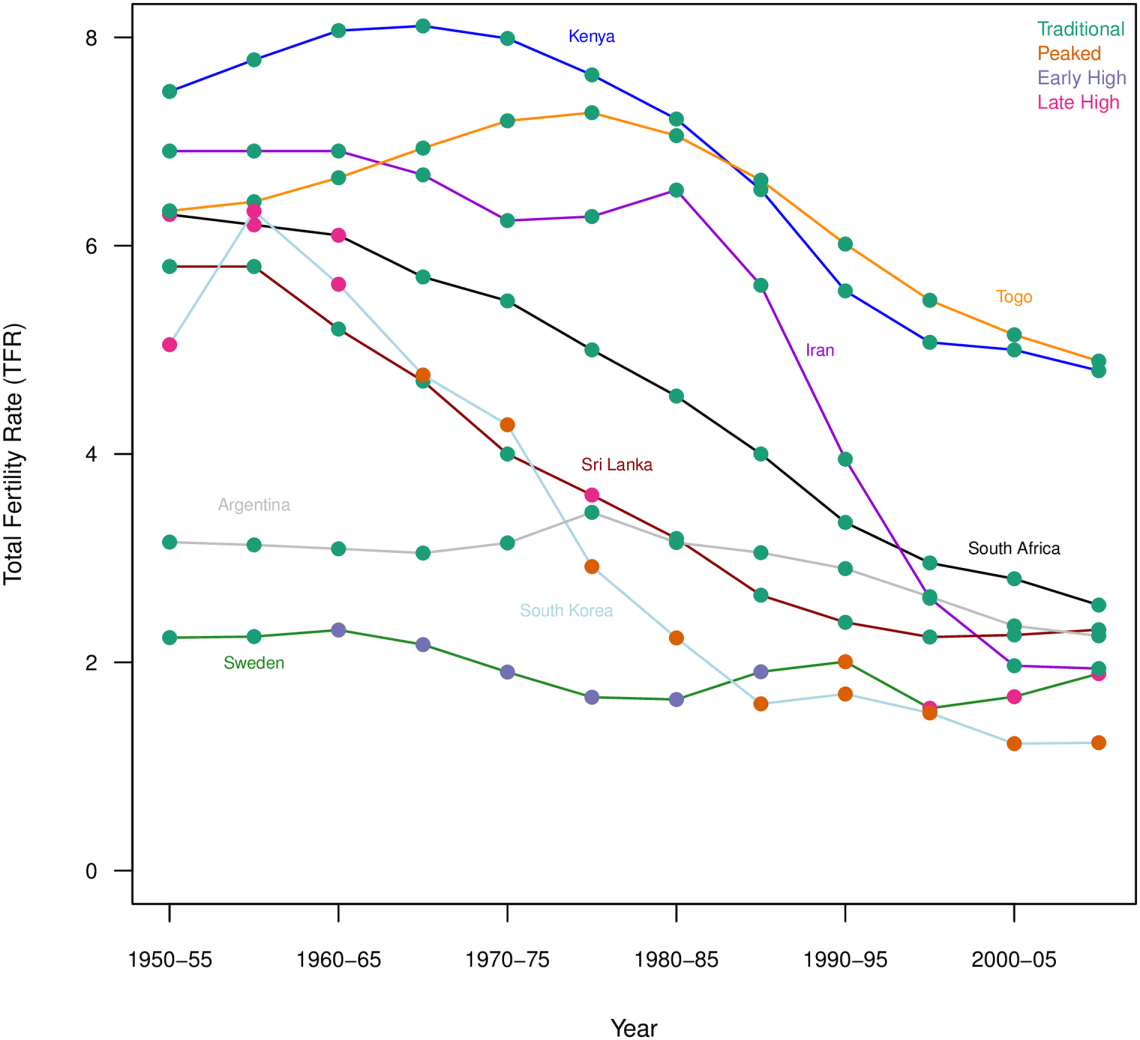
Trends in total fertility rates with cluster assignments for select countries.

Fig 9 shows the TFR trends for selected countries from 1950-55 through 2005-10 with the associated cluster membership shown for each period. Three countries from SSA are shown here, Kenya, Togo and South Africa, chosen because of their evident fertility decline in the past few decades. Kenya and Togo are in the Traditional cluster for all the periods shown here, indicating they kept a relatively stable age-specific fertility regime through their fertility declines. South Africa started in the Late High cluster in the initial periods covered but has been categorized as Traditional since 1965-70 and throughout its decline to near replacement fertility. This pattern is consistent with that seen for Iran, also shown, which had a precipitous decline without changing from its categorization in the Traditional cluster, indicating minimal changes to its age-specific fertility curve. Sri Lanka, as well, was categorized in the Traditional cluster throughout its fertility decline, except for one period (1975-80) that was categorized as Late High. Argentina’s fertility was largely stalled throughout the period under study with some decline to replacement levels seen towards the end of the study period; Argentina’s fertility curves were categorized as Traditional for all periods under study. South Korea and Sweden are included for comparison. South Korea is an example of a country with many Peaked fertility curves. Early South Korean curves were categorized as Late High and then during the decline changed to Peaked. Curves were categorized as Peaked through the decline to below replacement levels. Meanwhile curves from Sweden were included in all clusters. Curves from the first two periods were Traditional. Afterwards Swedish fertility saw a decline to below replacement levels during which the curves were categorized as Early High. One period was categorized as Peaked, followed by two periods categorized as Late High, and finally the curve for the last period was categorized as Traditional. These last fluctuations from cluster to cluster correspond to a period of volatile TFR as Sweden’s fertility rose above and then dropped below replacement levels.

Clearly, patterns of decline in SSA’s more advanced fertility declines show similarities to patterns seen in other regions, suggesting the fertility behaviors outlined in Caldwell’s African Exceptionalism will not necessarily keep SSA countries from seeing fertility decline similar to patterns seen in other countries, if not comparatively delayed. Instead, these similar patterns in the more advanced declines of SSA point to the interpretation of Bongaarts and Casterline [2] that SSA fertility decline remains early and suggesting that changes in fertility regimes will likely occur in recognizable patterns.

## Discussion

Incorporating age patterns of fertility directly in modeling provides more depth to the analysis of global fertility trends by making explicit how changes in fertility may occur within stable age patterns of fertility and also how changing age patterns of fertility may result in only small TFR changes in the short-term. This analysis makes explicit the idea that many different patterns of fertility can result in similar levels of fertility, or that different levels of fertility can have very similar age patterns. Seeing that no particular age pattern structure was associated with particular fertility levels supports Hirschman’s [24] observation that a high fertility regime may take many different pathways to arrive at lower fertility. By reducing the dimensionality of the fertility curves using the SVD, we are able to look at these patterns with few parameters without losing detail, which simplifies analysis. This simplification suggests immediate next steps, specifically utilizing these outputs to test directly how factors hypothesized to contribute to fertility decline are affecting both fertility level (the first component) and the age structure of fertility (the second and third components).

Our results still support the primacy of the overall level; the explanatory strength of first SVD component and the large membership of the Traditional cluster suggest that the general fertility curve is the most prevalent. However, from our clustering results we see distinctive patterning of differences in age-specific fertility curves that are not associated with fertility level. Categorizing the fertility regimes into clusters allows us to visualize how changes in the age structure of fertility are occurring differently across fertility declines. Our work suggests some regional patterning in how age-specific fertility changes during fertility decline. Looking at the weights on our SVD components and looking at the clustering results, we see very little change in age-specific fertility patterns in SSA. However, we also see virtually no change in these patterns in Latin America and the Caribbean, and for some Asian countries. Casterline and Odden [25] also find similarities in birth intervals between SSA countries, Latin America and the Caribbean countries and some Asian countries. Among these countries we see examples where countries have maintained a traditional age-specific fertility curve throughout fertility decline to replacement fertility levels, effectively experiencing decline at all ages more or less simultaneously. In addition, we see some Western countries returning to this general age-specific fertility curve as their fertility levels stabilize after falling below replacement fertility levels. Considering SSA fertility trends in their global context this way, the relative stability in age patterns of fertility is not necessarily an indicator that substantial fertility decline will not occur and, as seen in some countries in Latin America and the Caribbean this decline could be relatively sudden even without a change in age patterns of fertility. For example, South Africa and Kenya (Fig 9) are following trajectories of both fertility decline and cluster membership (indicating age-specific fertility behaviors) similar to those seen in countries like Iran and Sri Lanka, which have reached replacement or near replacement fertility levels in recent decades. Investigating not only how the TFR is changing over time globally, but also by understanding how the age patterns of fertility change during the decline, can be potentially useful for predicting subsequent age patterns of fertility and for exploring how factors influencing fertility decline, such as economic development, women’s education, infant and child mortality, HIV/AIDS, etc., are related to age-specific fertility patterns.

This analysis demonstrated that dimension reduction of age-specific fertility curves is possible and that with reduced dimensions a coherent story of historical trends can be visualized and analyzed. The Swedish example, with the high quality, annual data spanning over a century illustrates the level of detail in age-specific fertility schedules that can be retained in a fraction of the dimensions. In the global analysis, investigating the patterns of fertility decline and age-specific fertility concurrently allow us to compare patterns of age-specific fertility curves over time across countries, which provides a global context for fertility behaviors seen in individual countries or regions. Specifically, investigating how SSA age-specific fertility patterns fit into global patterns, we can see that for SSA countries with more advanced fertility declines, their patterns of age-specific fertility are similar to patterns found in other regions.

## References

[1] United Nations, Department of Economic and Social Affairs, Population Division. World Population Prospects: the 2015 Revision, Key Findings and Advance Tables; 2015.

[2] Bongaarts J, Casterline J. Fertility Transition: Is sub-Saharan Africa Different? Population and development review. 2013;38:153–168. doi: 10.1111/j.1728-4457.2013.00557.x 24812439 10.1111/j.1728-4457.2013.00557.x PMC4011385

[3] Caldwell JC, Orubuloye IO, Caldwell P. Fertility Decline in Africa: A New Type of Transition? Population and development review. 1992;18(2):211–242. doi: 10.2307/1973678

[4] Machiyama K. A re-examination of recent fertility declines in sub-Saharan Africa. MEASURE DHS; 2010. 68.

[5] Dorius SF. Global Demographic Convergence? A Reconsideration of Changing Intercountry Inequality in Fertility. Population and development review. 2008;34(3):519–537. doi: 10.1111/j.1728-4457.2008.00235.x

[6] United Nations. World Population Prospects: the 2012 Revision; 2013 Available from: http://esa.un.org/wpp/index.htm.

[7] Knodel J. Family limitation and the fertility transition: Evidence from the age patterns of fertility in Europe and Asia. Population Studies. 1977;31(2):219–249. doi: 10.2307/2173916 22077839 10.1080/00324728.1977.10410428

[8] Chackiel J, Schokolnik S. Latin America: Overview of the Fertility Transition, 1950-1990 In: Guzmán JM, editor. The fertility transition in Latin America. Oxford University Press, USA; 1996 p. 3–26.

[9] van de Walle E, Foster A. Fertility Decline in Africa: Assesment and Prospects. World Bank; 1990. 125.

[10] Moultrie TA, Sayi TS, Timæus IM. Birth intervals, postponement, and fertility decline in Africa: A new type of transition? Population studies. 2012;66(3):241–258. doi: 10.1080/00324728.2012.701660 22891624 10.1080/00324728.2012.701660

[11] INDEPTH Network [Prepared by Clark Samuel J]. 7. In: INDEPTH Network, editor. INDEPTH Mortality Patterns for Africa vol. 1 of Population and Health in Developing Countries. Ottawa: IDRC Press; 2002 p. 83–128.

[12] Clark SJ, Jasseh M, Punpuing S, Zulu E, Bawah A, Sankoh O. INDEPTH Model Life Tables 2.0. In: Annual Conference of the Population Association of America (PAA). Population Association of America (PAA); 2009.

[13] Clark SJ. A Singular Value Decomposition-based Factorization and Parsimonious Component Model of Demographic Quantities Correlated by Age: Predicting Complete Demographic Age Schedules with Few Parameters; 2015. arXiv preprint arXiv:1504.02057. Available from: https://arxiv.org/abs/1504.02057.

[14] Sharrow DJ, Clark SJ, Raftery AE. Modeling Age-Specific Mortality for Countries with Generalized HIV Epidemics. PloS one. 2014;9(5):e96447 doi: 10.1371/journal.pone.0096447 24853081 10.1371/journal.pone.0096447 PMC4031074

[15] Coale AJ, Trussell TJ. Model fertility schedules: variations in the age structure of childbearing in human populations. Population index. 1974; p. 185–258. doi: 10.2307/2733910 12333625

[16] Lee RD. Modeling and forecasting the time series of US fertility: Age distribution, range, and ultimate level. International Journal of Forecasting. 1993;9(2):187–202. doi: 10.1016/0169-2070(93)90004-7 12319552 10.1016/0169-2070(93)90004-7

[17] Hyndman RJ, Ullah MS. Robust forecasting of mortality and fertility rates: a functional data approach. Computational Statistics & Data Analysis. 2007;51(10):4942–4956. doi: 10.1016/j.csda.2006.07.028

[18] Bryant J. Theories of Fertility Decline and the Evidence from Development Indicators. Population and development review. 2007;33(1):101–127. doi: 10.1111/j.1728-4457.2007.00160.x

[19] Wilson C. On the scale of global demographic convergence 1950–2000. Population and Development Review. 2001;27(1):155–171. doi: 10.1111/j.1728-4457.2001.00155.x 18589488 10.1111/j.1728-4457.2001.00155.x

[20] Human Fertility Collection [HFC]; 2015 (Downloaded on January 15). Available from: http://www.humanfertility.org.

[21] Strang G. Introduction to Linear Algebra 4e. Wellesley-Cambridge Press; 2009.

[22] Golub GH, Hoffman A, Stewart GW. A generalization of the Eckart-Young-Mirsky matrix approximation theorem. Linear Algebra and Its Applications. 1987;88:317–327. doi: 10.1016/0024-3795(87)90114-5

[23] Fraley C, Raftery AE, Murphy TB, Scrucca L. mclust Version 4 for R: Normal Mixture Modeling for Model-Based Clustering, Classification, and Density Estimation. Department of Statistics, University of Washington; 2012 597.

[24] Hirschman C. Why fertility changes. Annual Review of Sociology. 1994; p. 203–233. doi: 10.1146/annurev.so.20.080194.001223 12318868 10.1146/annurev.so.20.080194.001223

[25] Casterline JB, Odden C. Exceptional or Global? Evidence for Postponement in the Developing World; 2016. Population Association of America Annual Meeting.

